# Disability and Living with HIV: Baseline from a Cohort of People on Long Term ART in South Africa

**DOI:** 10.1371/journal.pone.0143936

**Published:** 2015-12-01

**Authors:** Jill Hanass-Hancock, Hellen Myezwa, Bradley Carpenter

**Affiliations:** 1 Health Economics and HIV and AIDS Research Division (HEARD), University of KwaZulu-Natal, Durban, KwaZulu-Natal, South Africa; 2 Department of Physiotherapy, Faculty of Health Sciences, University of the Witwatersrand, Johannesburg, Gauteng, South Africa; University of Pennsylvania School of Medicine, UNITED STATES

## Abstract

**Background:**

Through access to life saving antiretroviral treatment (ART) in southern Africa, HIV has been reconceptualised as a chronic disease. This comes with new challenges of HIV-related co-morbidities and disabilities. We still lack an understanding of the types and scope of disabilities experienced by people on long term ART and how this impacts health, adherence, and livelihood. This paper describes the results of a cohort study examining the new health- and disability-related needs of the millions of people on ART in the region.

**Methods:**

Data was collected from a cohort of people who had been on ART for six months or longer in a semi-urban public health care setting in South Africa. 1042 adults (18 and older) participated in the cross-sectional study which investigated disabilities/activity limitations, health, ART adherence, depression symptoms, and livelihood. We analysed the associations between these constructs using descriptive statistics, and bivariate and multivariate analyses.

**Results:**

A large number of participants (35.5%) obtained a weighted score of two or more on the WHODAS 2.0 indicating possible activity limitations. A positive relationship was found between activity limitations and depression symptoms, adherence, and worse health outcomes, while none was found for BMI or CD4 count. These associations varied by type of activity limitations and, in some cases, by gender.

**Conclusion:**

Activity limitations are potentially experienced by a large portion of people on ART in southern Africa which impacts health and ART adherence negatively. These results highlight the importance of better understanding the new health-related needs of people who are on long term ART, as well as the nuances of the disability they experience. This is urgently needed in order to enable HIV-endemic countries to better prepare for the new health-related needs of the millions of people on ART in southern Africa.

## Introduction

Undoubtedly, HIV has been one of the most challenging health concerns in southern Africa. Free public access to lifesaving antiretroviral treatment (ART) has increased throughout the last ten years. In 2013, 7.6 million of the 21.2 million people living with HIV (PLHIV) who are eligible for treatment, were receiving ART in this region [1]. Through increased access to ART, AIDS-related deaths have declined [1], and people’s life expectancy and quality of life have improved [2, 3]. However, living with HIV in the long term comes with new experiences of diverse forms of disabilities and HIV-related co-morbidities [3–5].

Treatment itself does not fully reinstate health [3] and occurs alongside that of other co-morbidities (such as cardiovascular, kidney, and liver disease; osteopenia; cancer; neurocognitive diseases; mental health conditions and many others [3, 5–8]). Additionally, HIV affects healthy aging and is seen as leading to frailty [3, 9, 10]. Intense biomedical research has revealed that several inflammation-associated immunodeficiency complications and toxic effects are increased in those undergoing ART, calling for chronic care management of the disease [3, 6, 11]. This is further complicated by the fact that all antiretroviral drug regimens available in southern Africa can cause adverse effects of their own [12], potentially causing long term damage to body functions. Different drug regimens have different side effects. Meintjes review discusses these side effects extensively and list conditions such as nausea, headaches, renal failure, peripheral neuropathy, hypersensitivity, rashes, bone marrow suppression, hyper-glycaemia, hepatitis, anaemia, hyper-lactating and gastro-intentioned upsetting [12]. Although within the last year new drugs, who are said to have less side effects, have been introduced in the region it is most likely that patients will still present a complex picture of health related needs.

Clinicians in Africa are thus expected to manage this increased complexity of HIV as a chronic disease [3, 7]. Yet health care systems in southern Africa are still designed to provide acute HIV care and are not equipped to respond to the changed needs of the millions of patients on chronic care. Deeks et al. [3] argues that this change requires new skills in health care staff and a reshaping of health systems in these settings. This raises questions around what the health-related needs of PLHIV in the long term are and what skills health care workers in Africa need to acquire.

One astonishing aspect of the widely held discussions around Africa’s changed health care needs in the era of chronic HIV [3] is the lack of interest around the impact of disability [2, 5]. We currently promote ART both in order to achieve maximum therapeutic benefits and for treatment as prevention [13]. However, we only have a limited understanding of the prevalence and types of disabilities experienced by people on long term ART in the region and we have no understanding of how it impacts prevention, treatment, and livelihood in the long term. A small number of scholars have argued that if patterns mirror those of resource-rich settings, where ART has been available much longer, we will have to address the disabling effects of HIV on a large scale. Such a shift requires a re-conceptualisation of HIV and comprehensive care in the context of Africa [2, 5, 14–17]. While there is data on HIV-related disability available from resource-rich settings [15, 18, 19], as well as a number of publications on the intersection of HIV and mental health in Africa [7, 20, 21], only a few studies have used a disability lens to investigate the experience of PLHIV in Africa and even fewer have investigated this for people on ART [5].

**Fig 1:**
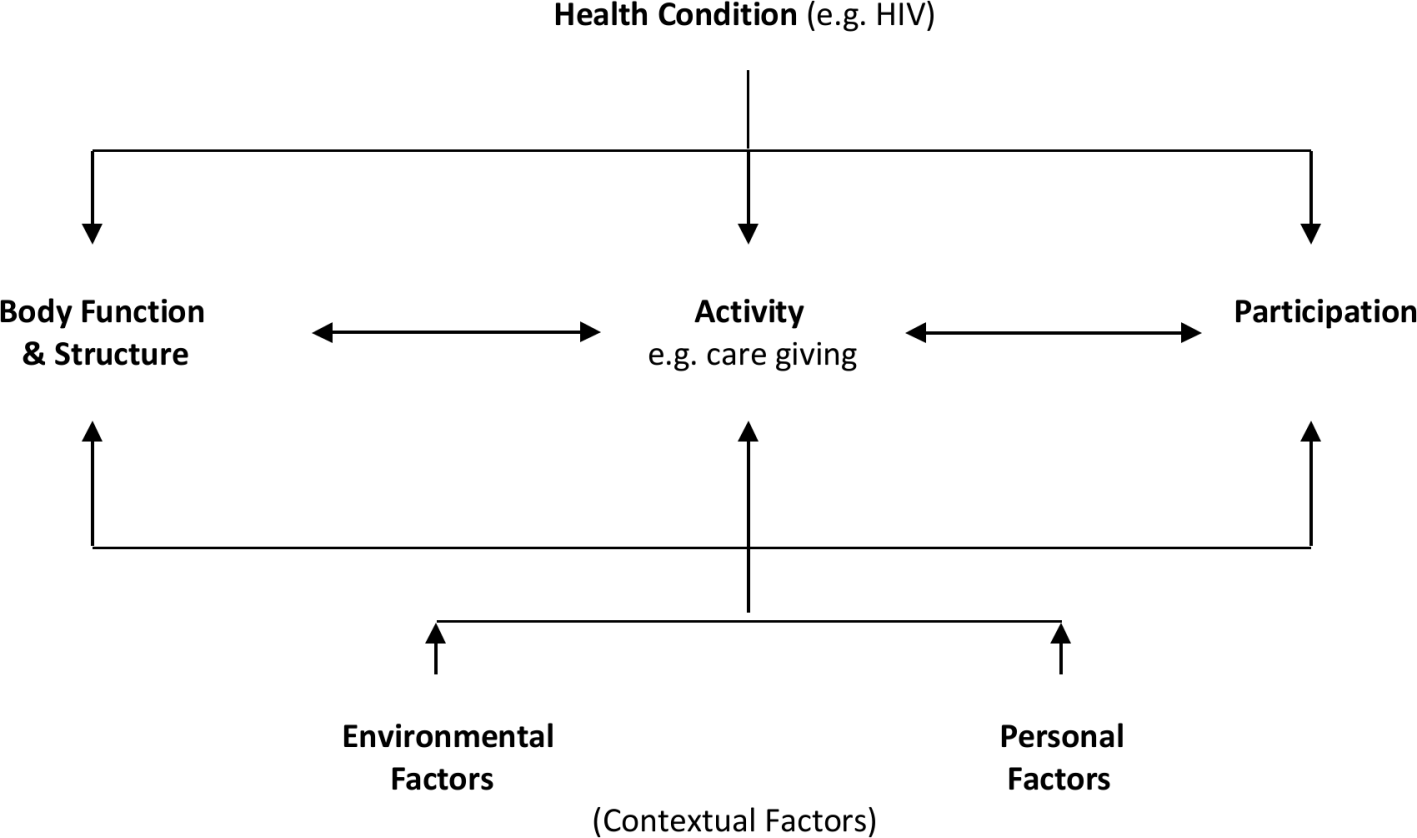
ICF model of function, disability and health.

The few existing studies suggest that disability is a potential risk for a significant portion of PLHIV, especially those on ART [14, 22, 23]. These studies often use the International Classification of Function, Disability and Health (Fig 1) as a guiding framework and lens to conceptualise disability. Within this framework health conditions such as HIV and its associated co-morbidities are understood to affect not only diverse body systems but also lead to permanent or episodic disability on body function (pain, blindness), activities (restrictions in walking and self-care), and participation (days-out of role, limited community participation). Furthermore, the ICF framework conceptually includes “contextual factors”, offering an understanding of how disability is shaped by inaccessible environments (e.g. physically inaccessible clinics or transport); personal factors (e.g. gender or resilience); and other social, political, and economic forces (e.g. stigma or poverty).

In one of the first studies in Africa, Myezwa et al. [14] used the ICF to map which domains of function, activity, and participation are the most affected. In three studies using small cohorts of patients (12–80) they found a significant number of patients experiencing impairments on a range of body functions (mental/cognitive, sensory, cardiovascular, respiratory, etc.). Limitations on the activity and participation levels were also found, with mobility, self-care, domestic activities, work, and community participation being some of the most affected domains. Similar issues were identified by Hanass-Hancock et al. [22] in a qualitative investigation into the experiences of disability in a cohort of people who had been on ART for six months or longer in KwaZulu-Natal, South Africa. The study posited a possible relationship between the development of mental health conditions and other impairments/activity limitations. This study potentially breaks new ground in understanding the nuances of mental health conditions in PLHIV and highlights the importance of multidisciplinary teams throughout the continuum of HIV care. In addition to this, participants rated activity limitations as being much more important than the actual health condition, as these were affecting their livelihoods and their participation in social life [22]. Furthermore, experiences of disability relating to gender norms and cultural context differed between men and women [22]. Men placed more importance on employment and women were more concerned about keeping stable relationships and being able to conduct daily household chores. Hence, while the diagnosis and treatment of health conditions are the clinicians’ focus, the effects of permanent limitations in daily activities are much more important to PLHIV on ART. While clinicians manage the former, the latter can only be mitigated through including rehabilitation strategies and providing accessible environments. This would constitute a major first step towards acquiring the “new set of skills” that health care workers in Africa need to acquire. These early studies provided a description of disability among PLHIV, including those on ART, however, their small scale or qualitative nature does not allow for any conclusions in regards to the scale and impact of disability within HIV management. More systematic and population-based research is needed to understand these dimensions.

Two recent systematic reviews provided a first look at the potential scale of disability. They revealed that most previous studies have predominately described various health conditions and potential types of impairments without actually referring directly to disability concepts [4, 5]. They also substantiated the hypothesis that disability is experienced by a large number of PLHIV (including those on ART) in Africa. The reviews highlight that we have little understanding of how disability affects the livelihoods of PLHIV and their adherence, as well as the implications for any efforts to extend treatment. Considering this lack of knowledge and understanding, it is not surprising that disability and rehabilitation services are neglected when countries with resource limitations have to make decisions that are based on priority setting and budget limitations [24]. Hence, in order to better inform policy and practice we need to identify the scale of HIV-related disability among people on ART and how it impacts the treatment of HIV, as well as the well-being and livelihoods of PLHIV.

Thus, this study sought to measure activity limitations (disability) and its associations with health, adherence, and livelihood indicators in two cohorts of patients who had been on ART for six months or longer. This paper provides a first indication of the potential scale and impact of disability among PLHIV who are on ART using data from the first setting. The data presented here focuses on the intersection of disability, health, and ART adherence.

## Methods

Data for this study was collected during the baseline study of the HIV-Live project (HIV, disability and livelihood project). This study was informed by a systematic review of HIV-related disabilities in hyper-endemic countries [5], as well as a series of small scale qualitative and quantitative pilot studies [14, 20–22, 25]. It was set in a semi-urban area in KwaZulu-Natal, South Africa, which has one of the highest rates of HIV prevalence in the world. As one arm of a multi-centre approach, it included 1042 randomly selected patients who were on ART in a semi urban public health setting in KwaZulu-Natal, South Africa. The setting included one hospital-based and three associated ART clinics in the community. Participants had to be 18 years or older, be on ART 6 month or longer, and not in a stage of any acute disease (e.g. TB). They were recruited during their routine visit at one of the four clinics.

Data collected in the survey included socio-demographic information; measures of health (health symptoms, comorbid TB or diabetes, CD4 count, and BMI), function/disability, mental health, adherence, livelihood (human, financial, social, natural, and physical), food security, and shocks; and exposure to potential rehabilitation interventions.

Disability was defined using the ICF as a conceptual framework. As previous studies indicated the importance of activity limitations within this framework we used a tool that could measure this outcome. Hence, activity limitations were measured using the 12-item WHODAS 2.0, a common measure used within disability literature and based on the ICF. Conceptualising disability within the ICF framework locates functioning and activity limitations as the central elements of measuring disability, as opposed to the diagnosis of health conditions or impairments. The WHODAS is a widely used tool [10, 26] which measures functioning/disability related to a range of key activities that are instrumental to daily life. The items prompt questions in relation to six domains: cognition (learning, concentrating), mobility (standing, walking), self-care (washing, getting dressed), getting along (maintaining friendships, dealing with people), life activities (work/school), and participation (joining community activities, emotional effects). These questions are specific in regards to the duration of activities and are weighted with regards to the difficulty of the task and the severity of the response. For instance, in order to score on the concentration item the respondent has to identify that he or she had problems concentrating for 10min in the last 30 days. Responses are then scored using a 5 point Likert-type scale (none, mild, moderate, severe and extreme/cannot do at all). The overall WHODAS score was established using item-response theory-based scoring as developed by the World Health Organisation (WHO) [27]. The analysis in this paper uses an overall weighted score ranging from 0–36 (with 36 being the lowest level of functioning after combining all the domains) and a percentage score (where 100 equals full functioning/no disability and 0 equals severe functional problems in all domains).

Mental health was measured by symptoms of depression using the Center for Epidemiologic Studies Short Depression Scale (CES-D 10). This validated screening tool is widely used, including in South Africa [28]. It includes a set of 10 questions that prompt whether certain feelings or behaviours have occurred within the past week. The questions are scored on a 4-point Likert-like scale ranging from rarely to most of the time. The scores (0–3) are summed and a score of 10 or more is considered to be at risk of depression [29, 30]. The analysis in this paper uses the overall score as well as a percentage score (with 0 indicating the most severe symptoms of depression and 100 indicating no symptoms of depression).

Adherence was measured in two ways, by Mannheimer’s CASE adherence index and by CD4 count. Mannheimer’s CASE adherence index [31] is an easy to administer set of questions that provides an alternative method for measuring ART adherence to the standard biomedical methods. It consists of a set of three items that have been validated and is used in South Africa [28]. Mannheimer reports that the index correlated strongly with the three-day self-reported adherence data and HIV outcome measures, including a 1-log decline in HIV RNA level and CD4 count. A score of less than 10 is considered as indicating problems with adherence. We used the overall score as well as a percentage score (with 0 indicating no adherence and 100 full adherence) for the analysis in this paper.

CD4 count was measured through self-report by the patient and extracted from the patient’s medical records. Height and weight were also measured by field workers in order to calculate the patient’s BMI, and a list of HIV-related symptoms and side effects was used to measure overall health. The list was based on work by Duran et al. [32] and includes a list of 16 symptoms commonly experienced by PLHIV such as memory loss, confusion, diarrhoea, breathlessness, fatigue, stomach pain, headaches, nausea, fever, changing taste, itching skin, muscle pain, heartburn, sore mouth, vomiting, and kidney stones. An overall score and a percentage score were used (100 indicating full health with no symptoms and 0 indicating the presence of all symptoms).

The analysis examined descriptive statistics and then used bivariate and multivariate analyses in order to establish associations between items. The variables for gender, age, and years on ART were examined and controlled for. Following this, categorical variables were calculated for Mannheimer’s adherence index and the CES-10 using cut-off points of 10 for both [30, 31]. Using these variables, the unadjusted and adjusted odds ratios were estimated in regards to increasing activity limitations from binary logistic regression models. The variables of gender, age, and months on ART were examined and controlled for in all multivariate analyses.

In addition, we divided the sample into a group that experienced limitations and those that did not, using the WHODAS weighted score. Using the same method as the disability index employed for establishing disability prevalence with the Washington set of questions [33], we considered participants scoring 0 or 1 on the WHODAS as not experiencing activity limitations, and hence disability. All participants who scored more than 2 on the WHODAS, which is at least two mild/moderate or one moderate/severe limitation (depending on weighting of item), were considered as experiencing activity limitations. We were interested to see how the two groups performed not only in regards to activity limitations but also in regards to depressive symptoms, adherence, overall health symptoms, and indicators such as the BMI and CD4 count.

All analyses were conducted in SPSS 22. Ethical clearance was provided by the hospital setting, the department of health, the University of KwaZulu-Natal, University Biomedical Research Ethics Committee (REF BE 275/13) and WITs Human Research Ethics Committee (Medical M131187). Individual participants were approached and recruited during their routine collection of ART at the local health clinic. However, this approach potentially excluded some people with more severe disabilities. Participation was voluntary and patients were provided with information on the nature of the study prior to their participation. Once they decided to participate, written informed consent was obtained from all participants. The interviews were conducted in private rooms in the clinics. Participants who screened positive for mental health or disability were referred to the appropriate health care staff.

## Results

The study recruited 684 female (65.6%) and 358 male (34.4%) participants. From the original 1050 participants four questionnaires had to be eliminated due to not meeting the requirements for participation and four questionnaires had to be eliminated due to missing data. The age ranged from 18 to 88 years with a mean of 38 years. Participants had been on ART between six months and 20.2 years (only 23 participants before 2005). The mean age of the people who did not experience activity limitations was 37.1 years and those who did 39.9 years. The main socio-demographic characteristic of the cohort and sub-groups are displayed in Table 1. The socio-demographic data of this sample revealed that the income of participants was on average R5111.54 for the last three months. The group of people experiencing limitations had lower income, earning on average R4615.03 as compared to the R5386.06 of those without functional limitations, but the difference was not significant due to the large amount of variance. However, after substituting more reasonable income values for very large ones, in order to reduce their influence on the data, significance was reached (*t*[882] = 2.0, *p* < .05). Only a small number of people in this cohort (20 participants) reported receiving a disability grant and these were mainly people who scored relatively low on the WHODAS, with 16 of the 20 scoring 3 or less and nine scoring 1 or less on the scale.

**Table 1:** Social-demographic information of participants.

Characteristics		Whole Cohort (n = 1042)	WHODAS 0–1 group (n = 672)	WHODAS ≥ 2 group (n = 370)
**Age**	(mean)	38.1	37.1	39.9
**Gender**	**Men**	358	237	121
	**Women**	684	435	249
**Marital Status**	**Never married/single**	785	507	278
	**Currently married**	130	88	42
	**Divorced/Widowed**	36	15	21
	**Cohabiting**	89	60	29
**Source of Income**	**Earned Income**	500	340	160
	**Other Income**	61	42	19
	**Disability Grant**	20	9	11
	**Other Grants**	403	251	152
	**None provided**	0	0	0
**Income**	(mean)	6657.78	7073.33	5919.02
**WHODAS weighted score**	(mean out of 36)^a^	1.6	0.2	4.1
**CES-10 Score**	(mean out of 40) ^a^	17.0	15.9	19.1
**Adherence Score**	(mean out of 16) ^b^	15.2	15.4	14.8
**Health Symptoms**	(mean out of 16) ^a^	4.1	3.4	5.4

^**a**^Higher scores indicate worse health outcome.

^**b**^Higher scores indicate better adherence.

**Fig 2:**
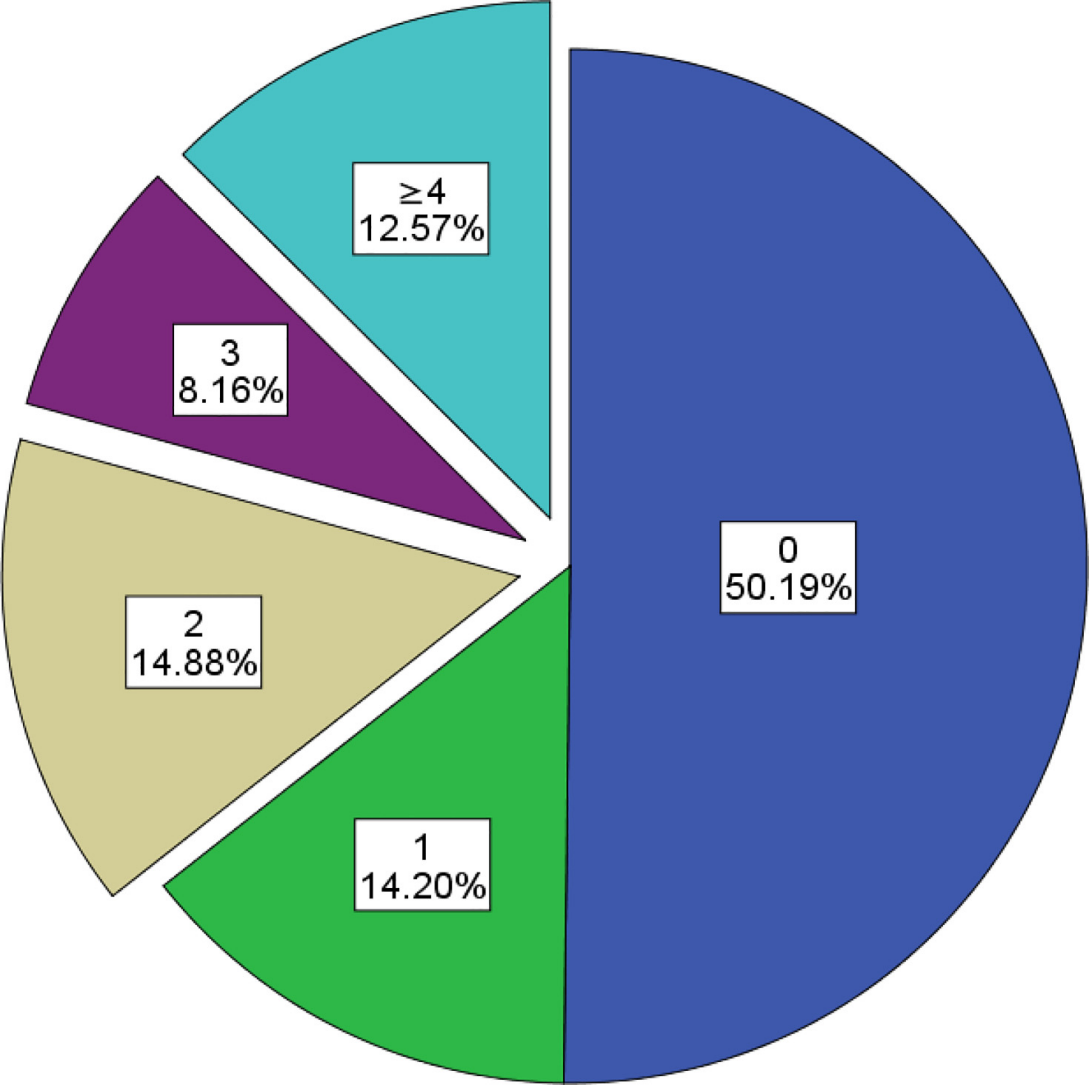
Percentage of Participants experiencing one or more activity limitations.

When analysing the overall cohort we found that activity limitations were experienced by a large number of people. Only 50.2% of participants did not score on the WHODAS scale at all. 14.2% of participants scored at a mild level on one limitation and 35.6% of the sample experienced at least moderate to severe activity limitations with two or more scores on the WHODAS (Fig 2). Further investigation of the WHODAS scores revealed that most activity limitations were moderate, with 64.7% of those experiencing limitations scoring 2 or 3 points on the WHODAS scale.

Correlation analysis showed a positive relationship between activity limitations and mental health (depression symptoms). This relationship held when controlling for other covariates like physical health symptoms. Additionally, it was found that this relationship is significantly stronger for women than it is for men (*r*_*τ*_ = .27, *p* < .001 and *r*_*τ*_ = .18, *p* < .001, respectively). The associations to depressive symptoms were particularly strong for certain domains of the WHODAS 2.0, such as mobility (*r*_*τ*_ = .22, *p* < .001), life activities (*r*_*τ*_ = .23, *p* < .001), and cognition (*r*_*τ*_ = .25, *p* < .001). Looking specifically at the groups with and without activity limitations, they averaged 19.1 and 15.9, respectively on the CES-D 10. Thus, the group experiencing activity limitations scored very close to the cut-off point, displaying a greater degree of depressed symptoms.

In the second part of this analysis we used logistic regression with continuous predictor variables in order to further investigate the strength of this relationship. It was found that with increasing limitations, the odds to screen positive for depressive symptoms increased 1.36 times (95% CI 1.28–1.46) for every additional point scored on the weighted WHODAS. Further investigation for different types of activity limitations revealed that this relationship depends on the WHODAS domain (see Table 2). For instance it was found that mobility (OR = 1.27, 95% CI 1.11–1.46), participation (OR = 1.55, 95% CI 1.27–1.89), life activities (OR = 1.86, 95% CI 1.18–2.92), and cognition (OR = 1.58, 95% CI 1.24–2.01) were all significant predictors of depression symptoms. However, life activities, participation, and cognition showed much stronger effects than mobility. Additionally, due to the high incidence of depressive symptoms in the entire sample (55.5%), the odds ratios were also converted into relative risk in order to correct for overestimation [34, 35]. This led to changes in the size of the estimates and to a change in terms of which were significant. Limitations of any type (RR = 1.12), mobility (RR = 1.13), cognition (RR = 1.19) and participation (RR = 1.20) remained predictors while life activities (RR = 1.07) became non-significant and getting along (RR = 0.81) became significant.

**Table 2:** Relative Risk Ratios.

Factor	Mental Health		Adherence	
	RR (Unadjusted) 95% CI	RR (Adjusted) 95% CI	RR (Unadjusted) 95% CI	RR (Adjusted) 95% CI
**Global Limitation**	**1.12 (1.10–1.14)**	**1.12 (1.09–1.14)**	**1.09 (1.05–1.14)**	**1.09 (1.05–1.14)**
**- Mobility**	**1.15 (1.07–1.23)**	**1.13 (1.06–1.21)**	**1.33 (1.16–1.53)**	**1.34 (1.16–1.55)**
**- Life Activity**	1.04 (0.87–1.25)	1.07 (0.90–1.27)	0.71 (0.44–1.16)	0.70 (0.42–1.15)
**- Cognition**	**1.22 (1.09–1.36)**	**1.19 (1.06–1.33)**	1.06 (0.79–1.43)	1.08 (0.79–1.46)
**- Participation**	**1.19 (1.08–1.31)**	**1.20 (1.09–1.33)**	1.15 (0.90–1.49)	1.15 (0.89–1.47)
**- Self-care**	0.85 (0.71–1.02)	0.88 (0.72–1.07)	0.66 (0.31–1.41)	0.66 (0.31–1.39)
**- Getting Along**	**0.80 (0.64–0.99)**	**0.81 (0.65–1.00)**	0.97 (0.65–1.44)	0.98 (0.65–1.47)

A positive association also emerged between activity limitations and the number of health-related symptoms experienced by participants, with increased functional limitation being linked to more health symptoms. As with mental health, physical health exhibited a significantly stronger relationship to activity limitations for women than for men, (*r*_*τ*_ = .28, *p* < .001 and *r*_*τ*_ = .16, *p* < .001, respectively).

Associations between activity limitations and adherence were less conclusive than the above, and more nuanced analysis was necessary. Adherence was in general very high in this cohort, with the lowest scores being among the group of 18–29 year olds. Both males and females showed high levels of adherence, with only 8.1% of males and 6.9% of females falling below the acceptable adherence line. Using the WHODAS weighted score a weak, negative association emerged between activity limitations and adherence, (*r*_*τ*_ = −.13, *p* < .001). Looking into this in more detail we found that the association between these limitations and adherence is only significant for women and does not hold for men (*r*_*τ*_ = −.16, *p* < .001 and *r*_*τ*_ = −.08, *p* > .05, respectively). Additionally, adherence was found to have a significant relationship with the domains of mobility (*r*_*τ*_ = −.15, *p* < .001) and community participation (*r*_*τ*_ = −.08, *p* < .01), although both are weak. Using regression analysis, adherence had a significant relationship with activity limitations (OR = 1.11, 95% CI 1.05–1.18) that was only explained through the domain of mobility (OR = 1.41, 95% CI 1.18–1.68). Hence, the relationship between adherence and activity limitations in general may be due almost entirely to mobility limitations. All other domains were insignificant. Similar results were found for relative risk ratio (see Table 2), although the value for relative risk is smaller than for the odds ratio.

There was also no association between activity limitations and BMI or CD4 count. This was found both in the original correlation and when controlling for gender, age and months on ART. Months on ART was also not associated to disability scores.

**Fig 3:**
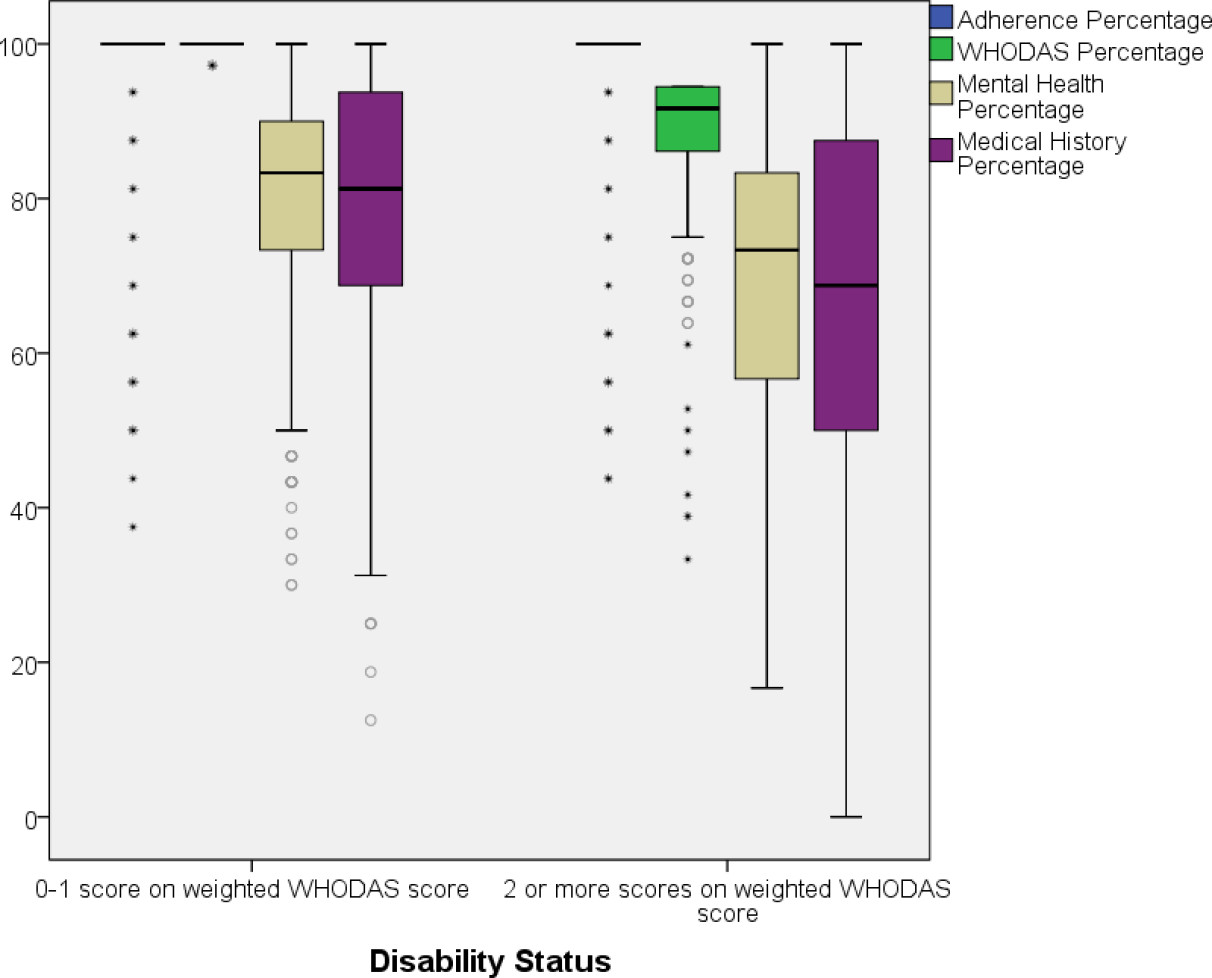
Boxplot of converted health scores in percentages split by disability status.

In a final step we compared the overall performance in terms of health status of the groups with (35.5%) and without activity limitations (64.5%). The 35.5% of the sample who recorded a score of 2 or more on the WHODAS 2.0 were considered as experiencing activity limitations, which could be considered to indicate disability. A comparison between the groups can be seen in Fig 3.

Overall the group experiencing activity limitations performed worst in most health measures (depression, health symptoms, and adherence). However, the groups were very similar in regards to measures such as BMI, age and CD4, indicating that the two groups were quite comparable in regards to current approaches of disease management. As a large number of participants in this sample experienced activity limitations and at the same time other health issues, this poses the question as to whether the health system is prepared to respond to these diverse needs.

## Discussion

The presented study indicates that a significant number of people on ART (35.5% in our cohort) experienced activity limitations which could be said to indicate the onset of disability. This percentage is much higher than the disability prevalence reported in national surveys in South Africa, which also use activity limitation questions as an indicator of disability. For instance, South Africa’s National Census in 2011 revealed a prevalence of 7.5% using a disability index based on the Washington set of questions [33, 36].

Our results raise two important issues. Firstly, the potentially large number of people on ART who experience disablement has serious implications for the fragile health and social systems in southern Africa. It also raises questions around disability prevalence among those on long-term ART in Africa and how it can be prevented or managed. As a first step, a comprehensive mapping of these disabilities and their impact is imperative. It is especially important that studies examining disability sample exhaustively to eliminate the possibility of excluding those with more severe disabilities, particularly when using populations already accessing health care facilities independently. The exclusion bias has the potential to underestimate the true impact of disability on PLHIV who are on ART.

Secondly, experiences of disability have become a common part of living with HIV in Africa [2, 4, 5, 14, 22, 37]{Banks, 2014 #4178} and therefore rehabilitation needs to become a crucial component of HIV treatment, care, and support [37]. However, while the global discourse around HIV has acknowledged the fact that HIV has become a chronic disease [3], it is still couched in a predominantly medical model. Discourses focus mainly on the medical treatment of co-morbidities and associated chronic care, ignoring the importance of interventions such as rehabilitation that are important in chronic care. The lack of focus on rehabilitation and quality of life in the response could be attributed to a lack of population-based disability related data and information, which suggests a need for more studies, in particular longitudinal studies that investigate the change over time in the experience of disability for people on ART.

Our study also indicates interlinkages between activity limitations and other health outcomes. The earlier studies of PLHIV on ART in Africa by Myezwa et al. and Hanass-Hancock et al. [14, 22] described the existence of disability and related impairments, activity limitations and participation restrictions. These studies also described the interrelationships between these constructs but were not able to test relationships with other health outcomes. This may seem intuitive, but within the literature focusing on Africa, HIV-related health outcomes are seldom discussed in relation to activity limitations or even disability. For instance, there is a rich body of literature on mental health and HIV [7, 38–40] as well as suitable interventions [21, 39]; however, there is no consideration of disability issues. In his systematic review Brandt [7] finds that on average half of the HIV-infected adults in Africa have some form of mental health problem with depression being the most common. The shared conclusion in these studies is a call for mental health interventions in the context of HIV [7, 20, 39], again, without any consideration of other disabilities at all. While there is strong evidence supporting the importance of mental health interventions, research in Africa so far has not investigated the relationship between activity limitations/participation restrictions (disability), and HIV-comorbid conditions such as depression and anxiety. Although this first cross-sectional study cannot explain causality, it needs to be noted that disability outside the field of HIV has been described as a driver of health disparities [41–45] Investigating the causality behind the relationship of mental health and disability could potentially have many implications for practice in the field.

In fact, our results suggest a strong association between measures of activity limitations and depression symptoms in people on ART. A more nuanced investigation revealed that this association was particularly strong for people who experience limitations in the domains of “mobility” and “cognition”, and less so for those who experience other limitations. This suggests that the loss of cognitive or mobility functions is increasing depressive symptoms, or that depressive symptoms lead to a skewed perception of the ability to perform these functions. In addition these findings may be contextualised association and other disability types may be associated with mental health problems in other settings. In order to determine impact and direction in future research into mental health and HIV, there should be more consideration around the role of disability as a mediating factor in depression. Vice versa, research on the intersection of disability and HIV needs to be cognisant of the effects that mental health conditions may have.

The analyses also revealed that the nuances of disability need to be fully considered when attempting to understand the impact of activity limitations on other outcomes. In a similar manner to mental health, our study found diverse results in regard to adherence outcomes with respect to the different domains of the WHODAS 2.0. The association between adherence and mobility suggests that people with mobility limitations in this sample experience barriers when accessing health care or that adherence issues are associated with developing mobility limitations. In fact, literature on people with disabilities and HIV suggests that mobility is a key issue when accessing health care. This is because health services and structurally related services such as transport are physically inaccessible to those with mobility limitations [46–48]. In addition, studies suggest that women with disabilities do not have the necessary support networks for adequate adherence as they more frequently report that they have been abandoned by their partners or families after disclosing their HIV or disability status [22, 49]. Such results suggest that barriers related to disability, as well as its intersection with other determinants of discrimination, need to be considered when investigating adherence to ART. This is particularly important as our results indicate no relationship between time on ART and activity limitations, suggesting that time on ART does not impact the experience or severity of disability. However, considering that people with disabilities are more likely to experience health disparities and are less likely to access health services, they may be more likely to drop out over time. As the high drop-out rates in ART programmes in Southern Africa are largely unexplained, it is likely that disability may play a significant role in adherence. If true, drop outs due to disability would mask the effect of increasing disability over time when using a cross-sectional design. Longitudinal and qualitative studies are necessary to investigate this further.

Lastly, our results have implications for policy and practice. South Africa’s current National Strategic Plan on HIV includes an objective relating to disability, namely: “Sustain health and wellness, primarily by reducing deaths and disability from HIV, AIDS and TB” [50]. However, the plan falls short in identifying which disabilities need to be reduced and how this “reduction” will be achieved. The most evident gap in the plan is the absence of any mention of rehabilitation services. However, the shift of HIV to a chronic illness in southern Africa requires a broadened approach and health services need to go beyond the acute biomedical responses. The rehabilitation framework offers just such an approach. HIV policy and programme documents need to better identify the disabilities amongst those living with HIV and provide guidance on how to integrate rehabilitation into the management of HIV as a chronic illness. Conversely, the rehabilitation fraternity needs to take cognisance of the impact of HIV on its profession in southern Africa. South Africa is currently reviewing its framework and strategy for disability and rehabilitation services. This strategy needs to speak to priority programmes such as HIV and AIDS, and identify how rehabilitation can offer a framework that addresses the disabling effects of HIV.

Integrating rehabilitation into HIV care (and vice versa) might not only be a question of policy and shifting of programme priorities, it might also be a question of feasibility and resources. One could argue that rehabilitation services are just too expensive or challenging for an already overburdened health system. However, research in South Africa has already piloted some interesting interventions that take into account the challenges of settings with limited resources. For instance, in the field of mental health the provision of depression interventions for PLHIV has been successfully piloted with a task shifting approach [21]. Furthermore, this approach is currently being taken forward in larger scale intervention trials [51, 52]. In addition, rehabilitation can also be used with a preventative framework ensuring that people at risk practise healthy habits that prevent the development of limitations [53].

With the exception of mental health, there is little evidence of rehabilitation interventions, and none on feasibility or costs. We need much more research to understand the potential impact, effectiveness, and cost-benefit of such interventions. Some scattered work on physiotherapy interventions in Africa has demonstrated their potentially positive impact on body fat distribution, adiposity, metabolic function, and quality of life [53–55]. Despite these pilot studies, operational research in South Africa suggests that clinic-based physiotherapy approaches do not necessarily reach PLHIV who experience disability. This is due to a number of financial and structural barriers that patients with activity limitations experience, such as a lack of access to public transport [56].

Hence, besides policy review we need to explore approaches focussing on community outreach in order to deliver care. Surprisingly, both the disability and HIV field have developed community outreach approaches parallel to one another. The field of disability has developed Community Based Rehabilitation (or in some cases Home Based Rehabilitation) [57–59], while the field of HIV has developed Home Based Care [17, 60]. Although these two approaches use similar definitions centred around care and rehabilitation, they address very different issues in practice and the two approaches have seldom been used in combination [17]. Yet in the light of reduced aid and resources it seems necessary to understand how these parallel systems can be integrated. Such an approach would be in line with recent efforts to integrate health care services and the general call for the provisioning of comprehensive care [3, 51].

Consequently the complexity of managing chronic HIV in the era of ART calls for a better understanding of disability in people on ART, the integration of rehabilitation into HIV care, but also for innovation in regards to prevention and identification of disability, and the provision of rehabilitation. The diversity in potential disease progression provides a particular challenge for fragile health systems which lack highly qualified health care staff [61, 62]. Hence, much more systematic research, including intervention research, is needed to understand how to respond to disability and the changing needs of patients on ART in southern Africa.

## Conclusion

Despite the advances of ART and improvements in quality of life of PLHIV in Africa, our data suggests that activity limitations, or disabilities, are more prevalent in people on long-term ART than previously anticipated. These limitations are interlinked with mental health outcomes as well as treatment adherence. The fact that 35.5% of our cohort experienced limitations with a score of two or more is alarming. It calls for a shift in our thinking of what is needed to provide comprehensive care for chronic HIV and how this can be delivered feasible to millions of people on ART in southern Africa. It also calls for prevention and rehabilitation to play a prominent role in HIV care, specifically with regards to its diverse approaches to therapy and community engagement.

Our data and analyses are also an appeal for more research, particularly in regards to understanding the diversity of disabilities and their impact on treatment adherence, other health outcomes, and livelihood over time. The study provides only a first look at these nuances and the specific results highlight the importance of the disaggregation of disability. Essentially, by clustering a range of very different experiences we may fail to see the scope and impact of disability. Additionally, we have not yet unpacked the true issues that underlie each specific experience of disability, and thus, are unable to offer adequate support.

Continued neglect of disability issues and the related nuances will lead us to ignore a significant public health concern in southern Africa. This may not only lead to other health issues and poorer adherence and dropouts from antiretroviral treatment, but it is also particularly important for development in those countries where HIV is endemic. In short, UNAIDS’ vision of 90:90:90 [48, 63] will not be achieved without addressing disability in the region. As the HIV epidemic progresses into a chronic illness, rehabilitation interventions need to become part of the standard package of care and support, not only to prevent or mitigate disability, but also to enable people on ART to build livelihoods and live a life with the greatest possible function and wellness. How this can be achieved in HIV endemic countries of southern Africa, which are also resource poor settings, is an urgent question that is yet to be addressed.
